# The inhibition of functional expression of calcium channels by prion protein demonstrates competition with α_2_δ for GPI-anchoring pathways

**DOI:** 10.1042/BJ20131405

**Published:** 2014-02-14

**Authors:** Anita Alvarez-Laviada, Ivan Kadurin, Assunta Senatore, Roberto Chiesa, Annette C. Dolphin

**Affiliations:** *Department of Neuroscience, Physiology and Pharmacology, University College London, London WC1E 6BT, U.K.; †Dulbecco Telethon Institute c/o Department of Neuroscience, IRCCS–Istituto di Ricerche Farmacologiche “Mario Negri”, Milano, Italy

**Keywords:** α_2_δ, auxiliary subunit, calcium channel, GPI anchor, prion protein, Ca_V_, voltage-gated Ca^2+^, DRM, detergent-resistant membrane, GPI, glycosylphosphatidylinositol, HA, haemagglutinin, KO, knockout, MBS, Mes-buffered saline, PI-PLC, phosphatidylinositol-specific phospholipase C, PNGase F, peptide N-glycosidase F, PrP, prion protein, PrP^C^, normal cellular PrP, PrP^Sc^, PrP scrapie, WT, wild-type

## Abstract

It has been shown recently that PrP (prion protein) and the calcium channel auxiliary α_2_δ subunits interact in neurons and expression systems [Senatore, Colleoni, Verderio, Restelli, Morini, Condliffe, Bertani, Mantovani, Canovi, Micotti, Forloni, Dolphin, Matteoli, Gobbi and Chiesa (2012) Neuron **74**, 300–313]. In the present study we examined whether there was an effect of PrP on calcium currents. We have shown that when PrP is co-expressed with calcium channels formed from Ca_V_2.1/β and α_2_δ-1 or α_2_δ-2, there is a consistent decrease in calcium current density. This reduction was absent when a PrP construct was used lacking its GPI (glycosylphosphatidylinositol) anchor. We have reported previously that α_2_δ subunits are able to form GPI-anchored proteins [Davies, Kadurin, Alvarez-Laviada, Douglas, Nieto-Rostro, Bauer, Pratt and Dolphin (2010) Proc. Natl. Acad. Sci. U.S.A. **107**, 1654–1659] and show further evidence in the present paper. We have characterized recently a C-terminally truncated α_2_δ-1 construct, α_2_δ-1ΔC, and found that, despite loss of its membrane anchor, it still shows a partial ability to increase calcium currents [Kadurin, Alvarez-Laviada, Ng, Walker-Gray, D’Arco, Fadel, Pratt and Dolphin (2012) J. Biol. Chem. **1287**, 33554–33566]. We now find that PrP does not inhibit Ca_V_2.1/β currents formed with α_2_δ-1ΔC, rather than α_2_δ-1. It is possible that PrP and α_2_δ-1 compete for GPI-anchor intermediates or trafficking pathways, or that interaction between PrP and α_2_δ-1 requires association in cholesterol-rich membrane microdomains. Our additional finding that Ca_V_2.1/β1b/α_2_δ-1 currents were inhibited by GPI–GFP, but not cytosolic GFP, indicates that competition for limited GPI-anchor intermediates or trafficking pathways may be involved in PrP suppression of α_2_δ subunit function.

## Abstract 2

PrP, but not GPI-anchorless PrP, suppresses Ca_V_2.1/β/α_2_δ-1 or α_2_δ-2 calcium channel currents. However, PrP does not inhibit Ca_V_2.1/β currents formed with anchorless α_2_δ-1, rather than full-length α_2_δ-1. The results of the present study suggest that α_2_δ subunits and PrP may compete for GPI-anchor pathways.

## INTRODUCTION

Ca_V_ (voltage-gated Ca^2+^) channels contain a pore-forming α_1_ subunit, which determines the main properties, both biophysical and pharmacological, of the channels. For the Ca_V_1 and Ca_V_2 subfamilies, the α_1_ subunit is associated with a membrane-anchored predominantly extracellular α_2_δ subunit (for a review, see [1]) and a cytoplasmic β subunit (for reviews, see [2,3]). The β subunits have been shown to enhance calcium channel trafficking by binding to the I–II linker of the α_1_ subunits [4,5], and thus inhibiting proteasomal decay as has been shown for the Ca_V_2.2 [6] and Ca_V_1.2 [7] channels. In contrast, the mechanism of enhancement of the Ca_V_ channel functional expression by α_2_δ subunits has not been firmly established, although we have found that it involves their von Willebrand Factor A domain [8], and results in increased plasma membrane expression and active zone targeting of the channel complex [8,9].

The major mechanism whereby α_2_δ subunits increase the functional expression of calcium channels is due to an increase in the amount of channel protein at the plasma membrane ([8,10] and J.S. Cassidy and A.C. Dolphin, unpublished work). Furthermore, it has been found that the α_2_δ subunits do not increase single channel conductance or open probability [11,12], two other mechanisms whereby macroscopic current could be increased. Nevertheless, there are effects of α_2_δ subunits to increase voltage-dependent inactivation and to hyperpolarize the voltage-dependence of steady-state inactivation, providing evidence that these subunits do remain associated with the functional channel complex on the plasma membrane [8,13,14].

Mammalian genes encoding four α_2_δ subunits have been identified (for reviews, see [3,15]). The topology of the α_2_δ protein was first determined for α_2_δ-1, and is thought to generalize to all four α_2_δ subunits (for reviews, see [1,16]). They all have predicted N-terminal signal sequences, indicating that the N-terminus is extracellular. The α_2_ and δ subunits are the product of a single gene, encoding the α_2_δ pre-protein, which is post-translationally cleaved into α_2_ and δ [17]. The extracellular α_2_ subunit then remains disulfide-bonded to the membrane-bound δ subunit. Although α_2_δ subunits were originally described as transmembrane proteins, we have shown previously that they can form GPI (glycosylphosphatidylinositol)-anchored proteins [13].

The PrP^C^ [normal cellular PrP (prion protein)] is a GPI-anchored sialoglycoprotein [18] exposed on the outer leaflet of the plasma membrane, that is widely distributed throughout the nervous system [19,20]. Its normal physiological function is still being elucidated [20] and genetic ablation of PrP KO (knockout) only produces subtle phenotypes in mice [21,22]. However, it appears to play a role in signal transduction [23], copper binding [24], synaptic function [25] and myelin maintenance [26]. Furthermore, misfolding of PrP^C^ results in the formation of the partially protease-resistant isoform PrP^Sc^ (PrP scrapie), which accumulates in the brain and results in neurodegeneration, giving rise to Creutzfeldt–Jakob disease [19,27]. PrP^Sc^ is both neurotoxic and infectious. In the absence of PrP^C^, PrP^Sc^ cannot be generated and PrP-null mice do not propagate infectivity or develop pathology on infection with PrP^Sc^ [28–30]. Thus it has been postulated that the presence of PrP^C^ in neurons is essential for the initiation of a cascade of signalling events leading to prion disease pathology.

Initial evidence that the α_2_δ subunits are closely associated with PrP came from the finding that the α_2_δ-1, α_2_δ-2 and α_2_δ-3 subunits were co-immunopurified with PrP from the brains of transgenic mice expressing Myc-tagged PrP [31]. The α_2_δ subunits are expressed strongly in cholesterol-rich detergent-resistant membranes [13,32], as is PrP [33,34], which may represent the basis for their association. It has been shown recently that α_2_δ-1 and PrP can co-immunoprecipitate from both brain tissue and cell lines [35], and that the interaction between pathogenic mutant PrP and α_2_δ-1 results in the intracellular retention of both species [35].

We were interested to determine whether PrP co-expression has any direct effect on calcium channel currents via a direct or indirect effect on α_2_δ subunit processing or function. We therefore utilized full-length and C-terminally truncated PrP and α_2_δ-1 to examine the effect of these species on calcium channel functional expression.

## MATERIALS AND METHODS

### Heterologous expression of cDNAs

The calcium channel cDNAs used were rat Ca_V_2.1 (GenBank® accession number M64373), mouse α_2_δ-2 (GenBank® accession number AF247139), rat α_2_δ-1 (GenBank® accession number M86621), α_2_δ-1 mid HA (haemagglutinin) [36], α_2_δ-1ΔC–HA [36], rat β4 (GenBank® accession number NM001105733) and rat β1b [37]. The PrP constructs used (in pcDNA3) were WT (wild-type) PrP (mouse) and ΔGPI–PrP [38]. The GFP constructs used were mut3 GFP [39] and GPI–GFP in pcDNA3 [40]. The cDNAs were in the pMT2 expression vector, unless stated above. Mammalian tsA-201 cells were transfected with the following cDNA combinations (Ca_v_2.1/β1b/α_2_δ-1/PrP at 3:2:2:0.5 or Ca_v_2.1/β4/α_2_δ-2/PrP at 3:2:2:1), with empty vector replacing constructs not used unless otherwise stated, and transfection was performed as described previously [41]. The cDNA for GFP was also included to identify transfected cells from which electrophysiological recordings and imaging were performed, unless GPI–GFP was used.

### Mice

Cerebella were obtained from C57BL/6J (WT) mice or from Tg(WT-E1) mice expressing WT mouse PrP with an epitope for the monoclonal antibody 3F4 at approximately 4-fold the endogenous PrP level, referred to throughout the text as PrP Tg(WT) [42], or from PrP-KO mice with a pure C57BL/6J background (European Mouse Mutant Archive, Monterotondo, Rome; EM:01723) [21].

### Preparation of Triton X-100-insoluble membrane fractions [DRMs (detergent-resistant membrane)]

All steps were performed on ice. One cerebellum was used as the starting material from WT, PrP Tg(WT) and PrP-KO mice. The cerebella were homogenized using a Teflon homogenizer in MBS [Mes-buffered saline; 25 mM Mes (pH 6.5), 150 mM NaCl and Complete™ protease inhibitor cocktail (Roche)], containing 1% (v/v) Triton X-100 (Thermo Scientific), and left on ice for 1 h. An equal volume of 90% (w/v) sucrose in MBS was then added. The sample was transferred into a 13 ml ultracentrifuge tube and overlaid with 10 ml of discontinuous sucrose gradient, consisting of 35% (w/v) sucrose in MBS (5 ml) and 5% (w/v) sucrose in MBS (5 ml). The sucrose gradients were centrifuged at 138000 ***g*** for 18 h at 4°C (Beckman SW40 rotor). Fractions (1 ml) were subsequently harvested from the top to the bottom of the tube. When necessary, protein fractions from the gradient were washed free of sucrose by dilution in 25 volumes of ice-cold PBS and ultracentrifugation (150000 ***g*** for 1 h at 4°C) to pellet the cholesterol-enriched microdomain material. Triton X-100-insoluble protein was resuspended in deglycosylation buffer and treated with PNGase F (peptide N-glycosidase F; Roche), as described below.

### Treatment of Triton X-100-insoluble protein fractions with PI-PLC (phosphatidylinositol-specific phospholipase C)

Triton X-100-insoluble fractions from brain tissue were collected, washed free of sucrose and centrifuged as described above. The resultant pellet of Triton X-100-insoluble material was resuspended in an appropriate volume of PI-PLC reaction buffer [10 mM Tris/HCl (pH 7.4) and 150 mM NaCl containing Complete™ protease inhibitor cocktail (Roche)], to a final protein concentration of ~2 mg/ml. The samples were sonicated and treated with 25 units of PI-PLC enzyme (Sigma) for 3 h at 37°C.

### Phase separation of PI-PLC-treated proteins using Triton X-114

Membrane-associated proteins were separated from soluble proteins in two phases of Triton X-114 as described previously [43]. Briefly, the pellet of detergent-insoluble material was resuspended in an appropriate volume of reaction buffer (final concentration of ~2 mg/ml of protein) and incubated with PI-PLC as described above. Control experiments omitting the enzyme were also performed. After PI-PLC incubation the samples were supplemented with Triton X-114 (Thermo Scientific) to a final concentration of 1%. A cushion of 6% (w/v) sucrose, 10 mM Tris/HCl (pH 7.4), 150 mM NaCl and 0.06% Triton X-114 was placed at the bottom of a 1.5 ml Eppendorf tube. The protein sample was then overlaid on this sucrose cushion and the tube incubated for 3 min at 30°C and centrifuged at 300 ***g*** for 4 min at room temperature (20°C) in a swinging bucket rotor. Following centrifugation, the detergent phase was present as an oily droplet at the bottom of the tube. Fresh Triton X-114 was then added to the upper aqueous phase to 0.5% and the procedure was repeated using the same sucrose cushion. In the last step the aqueous phase was removed from the cushion, supplemented with fresh Triton X-114 to 2% and subjected to another centrifugation. The detergent phase of this last procedure was discarded. The aqueous and detergent phases from this procedure were adjusted to the equal volume with 10 mM Tris/HCl (pH 7.4) and 150 mM NaCl plus protease inhibitors.

### Acetone precipitation and PNGase F deglycosylation

To remove the remaining Triton X-114 in the detergent and aqueous phases from the phase separation experiment the proteins were precipitated by the addition of 4 volumes of ice-cold acetone and subsequent incubation for 1 h at −20°C. The precipitated material was centrifuged at 16000 ***g*** for 10 min and the pellet was washed once with an acetone/water (4:1) mixture (−20°C). The pellets of the precipitated proteins were then resuspended in 45 μl of PNGase F buffer [10 mM Tris/HCl (pH 7.5) and 150 mM NaCl supplemented with 75 mM 2-mercaptoethanol, 0.5% Triton X-100, 0.1% SDS and protease inhibitors]. A total of 1 unit of PNGase F was added per 10 μl volume followed by incubation at 37°C for 5–12 h. The samples were then resuspended in an appropriate volume of SDS gel loading buffer and heated for 10 min at 56°C in order to terminate the reaction.

### Immunoblotting

Western blotting was performed as described previously [41]. The samples were resolved on either 3–8% Tris/acetate or 4–12% Bis-Tris gels with the relevant buffer systems (Life Technologies). The samples were then blotted on to PVDF membranes (Bio-Rad Laboratories), blocked with 3% BSA in TBS provided with 0.5% Igepal and incubated with the following primary antibodies: anti-α_2_-1 (1:1000 dilution; mouse monoclonal; Sigma), anti-α_2_-2 (residues 102–117; rabbit polyclonal; [44]), anti-PrP (3F4 epitope), anti-PrP (residues 45–66; rabbit polyclonal; [45]) and anti-flotillin-1 (1:2000 dilution; mouse monoclonal; BD Biosciences). The secondary antibodies used were goat anti-mouse and goat anti-rabbit coupled to HRP (horseradish peroxidase; Bio-Rad Laboratories).

### Electrophysiology

Calcium channel expression in tsA-201 cells was investigated by whole-cell patch-clamp recording essentially as described previously [46]. The internal (pipette) and external solutions and recording techniques were similar to those described previously [47]. The patch pipette solution contained: 140 mM caesium aspartate, 5 mM EGTA, 2 mM MgCl_2_, 0.1 mM CaCl_2_, 2 mM K_2_-ATP and 10 mM Hepes, pH 7.2 at 310 mM mOsm, with sucrose. The external solution for recording Ba^2+^ currents contained: 150 mM tetraethylammonium Br, 3 mM KCl, 1 mM NaHCO_3_, 1 mM MgCl_2_, 10 mM Hepes, 4 mM glucose and 1 mM BaCl_2_, pH 7.4 at 320 mOsm, with sucrose. Pipettes of resistance 2–4 MΩ were used. An Axopatch 1D amplifier (Axon Instruments) was used, and data were filtered at 1–2 kHz and digitized at 5–10 kHz. Current records were subjected to leak and residual capacitance current subtraction (P/8 protocol). Analysis was performed using Pclamp 9 (Molecular Devices) and Origin 7 (Microcal Origin). All comparisons between different groups of transfected cells were performed in parallel on the same experimental days.

When stated, current–voltage (*I*–*V*) plots were fit with a modified Boltzmann equation for determination of the voltage for 50% activation (*V*_50_, activation) [48], and steady-state inactivation curves were fit with a single Boltzmann function for determination of the voltage for 50% inactivation (*V*_50_, inactivation). Where data are given as means±S.E.M., statistical comparisons were performed using either Student's *t* test or ANOVA with the appropriate post-hoc test.

*Xenopus* oocytes were prepared, injected and utilized for electrophysiology as described previously [49], with the following exceptions. Plasmid cDNAs for the calcium channel α1, α_2_δ-1 and β1b subunits and for PrP were mixed at 2:1:2:x ratios at 1 μg/μl, where x=2, 1 or 0.5 unless otherwise stated, and the empty vector was included to maintain the total cDNA mixture constant. A 9 nl volume was injected intranuclearly, after 2-fold dilution of the cDNA mixtures. Recordings in *Xenopus* oocytes were performed at 18°C as described previously [48], and all recordings were performed 48–60 h after injection for Ca_V_2.2. The Ba^2+^ concentration was 10 mM.

## RESULTS

### α_2_δ subunits and PrP show similar biochemical properties

**Figure 1:**
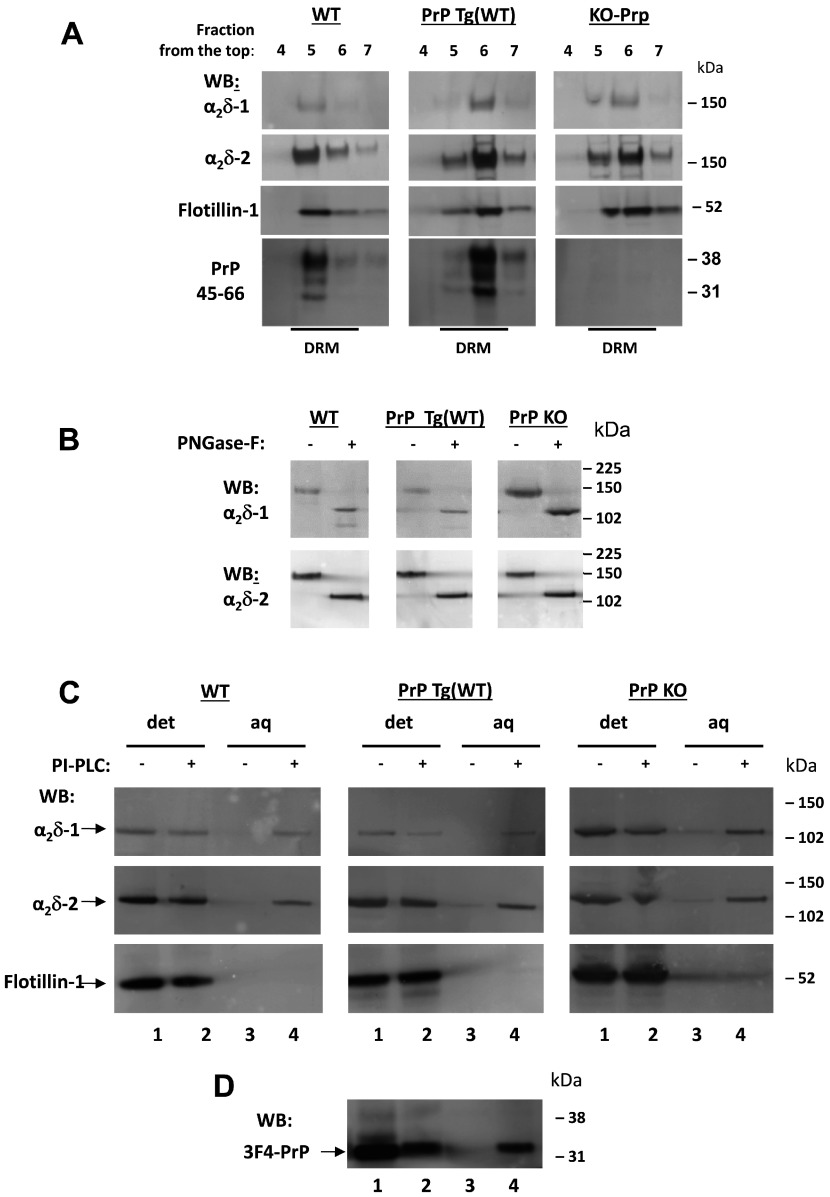
PI-PLC treatment and phase separation of α_2_δ-1, α_2_δ-2 and PrP in mouse cerebella (**A**) DRM fractions from WT (left-hand panel), PrP Tg(WT) (middle panel) and PrP KO (right-hand panel) mouse cerebella were prepared as described in the Materials and methods section. Aliquots were resolved on 3–8% Tris/acetate (to resolve α_2_δs) or 4–12% Bis-Tris gels (to resolve PrP in the same samples), and analysed by Western blotting (WB) with relevant antibodies as indicated. The full profile is not shown, but only the fractions of the sucrose gradient corresponding to DRMs identified by the presence of flotillin-1 (fractions 4–7 harvested from the top). The anti-α_2_δ-1 and anti-α_2_δ-2 antibodies recognize the α_2_-1 and α_2_-2 moieties. (**B**) Aliquots of concentrated DRM fractions from WT (left-hand panel), PrP Tg(WT) (middle panel) and PrP KO (right-hand panel) cerebella were treated with PNGase F and analysed by Western blotting with the indicated antibodies. (**C**) DRM fractions analysed in (**A**) were subjected to PI-PLC treatment and Triton X-114 phase separation (see the Materials and methods section), followed by PNGase F deglycosylation. The proteins remaining in the aqueous (aq) and detergent (det) phase were then resolved on 4–12% Bis-Tris gels and analysed with the indicated antibodies. Lanes 1 and 2 in each panel are from detergent phase fractions, whereas lanes 3 and 4 are from the respective aqueous phase, treated or not with PI-PLC as indicated. (**D**) The PrP Tg(WT) fractions from (**C**, middle panel) were also blotted for PrP using the 3F4 antibody. Molecular mass is shown on the right-hand side of the gels in kDa.

We have shown previously that endogenous α_2_δ-1 and α_2_δ-2 are strongly expressed in DRM fractions [13,32], as is PrP [18]. Therefore we prepared DRM fractions from the cerebella of WT, PrP Tg(WT) and PrP-KO mice. We found the typical localization of α_2_δ-1, α_2_δ-2 and also PrP in DRMs (Figure 1A).

It is well-established that PrP is GPI-anchored [18], and our previous findings indicate that the α_2_δ subunits can form GPI-anchored proteins [13]. One important piece of evidence supporting this premise is that following PI-PLC treatment, both endogenous α_2_δ subunits from the rat brain and heterologously expressed α_2_δ proteins redistribute from the detergent phase into the aqueous phase upon Triton X-114 phase separation [13]. This demonstrates that PI-PLC treatment has converted the protein into a hydrophilic species, most probably by removing the bulky hydrophobic GPI anchor. To assess if the behaviour of α_2_δ proteins in this phase-separation assay was affected by the presence or absence of PrP, we concentrated the DRM fractions enriched in both PrP and α_2_δ and subjected them to PI-PLC treatment as described previously [13]. An aliquot of the initial DRM material from the three mouse genotypes, before PI-PLC treatment, was deglycosylated with PNGase F to demonstrate the presence of α_2_δ-1 and α_2_δ-2 (Figure 1B).

PI-PLC treatment caused α_2_δ-1 and α_2_δ-2 to redistribute into the aqueous phase upon Triton X-114 phase separation in all three of the mouse genotypes tested (Figure 1C). As a control, the PrP Tg(WT) samples were also blotted for PrP, and redistributed similarly upon PI-PLC treatment (Figure 1D).

Importantly, the response of α_2_δ-1 and α_2_δ-2 to PI-PLC treatment was not affected by the absence of PrP, in the PrP KO cerebellar material (Figure 1C). It was also not affected by the overexpression of PrP in the PrP Tg(WT) cerebellar material (Figure 1C). These data indicate that although PrP has been reported to interact with α_2_δ subunits [35], interaction with PrP was not responsible for the presence of α_2_δ subunits in DRM fractions or their redistribution into the aqueous phase following PI-PLC cleavage of GPI-anchor sites.

### Co-expression of PrP decreased calcium channel currents containing Ca_V_2.1/β4/α_2_δ-2

**Figure 2:**
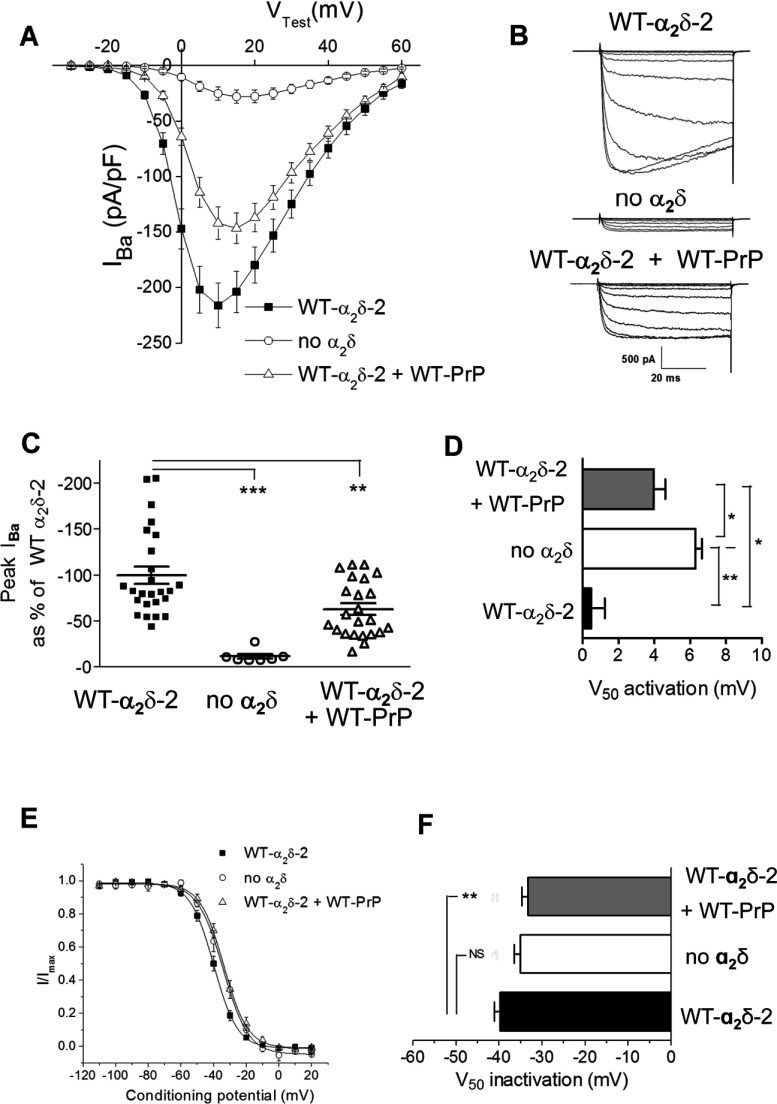
Effect of PrP on Ca_V_2.1/β4/α_2_δ-2 calcium channel currents (**A**) Current–voltage (*I*–*V*) relationships for *I*_Ba_ recorded from tsA-201 cells expressing Ca_V_2.1/β4/α_2_δ-2 (■; *n*=25), Ca_V_2.1/β4 alone (○; *n*=7) and Ca_V_2.1/β4/α_2_δ-2/WT PrP (△; *n*=23). The ratio of cDNAs used for transfection for Ca_V_2.1/β4/α_2_δ-2/WT-PrP was 3:2:2:1, with empty vector used where α_2_δ or PrP was absent. (**B**) Examples of families of *I*_Ba_ current traces resulting from step potentials from −100 mV to between −30 and +15 mV in 5 mV increments for Ca_V_2.1/β4/α_2_δ-2 (top panel), Ca_V_2.1/β4 alone (middle panel) and Ca_V_2.1/β4/α_2_δ-2/WT PrP (bottom panel). (**C**) Individual peak *I*_Ba_ currents at +10 mV, expressed as the mean±S.E.M. percentage of the control condition with WT α_2_δ-2 and Ca_V_2.1/β4/α_2_δ-2 (■; *n*=25), Ca_V_2.1/β4 alone (○; *n*=7) and Ca_V_2.1/β4/α_2_δ-2/WT PrP (△; *n*=23). **P<0.01 and ***P<0.001. (**D**) Voltage-dependence of activation (*V*_50_ activation) determined by fitting a modified Boltzmann function to the individual *I*–*V* relationships shown in (**A**) for Ca_V_2.1/β4/α_2_δ-2 (black bar), Ca_V_2.1/β4 alone (white bar) and Ca_V_2.1/β4/α_2_δ-2/WT PrP (grey bar). *P<0.05 and **P<0.01. (**E**) Steady-state inactivation curves for *I*_Ba_ recorded from cells expressing Ca_V_2.1/β4/α_2_δ-2 (■; *n*=12), Ca_V_2.1/β4 alone (○; *n*=4) and Ca_V_2.1/β4/α_2_δ-2/WT PrP (△; *n*=8). (**F**) Voltage-dependence of steady-state inactivation (*V*_50_ inactivation) determined by fitting a Boltzmann function to the individual steady-state inactivation relationships for the data shown in (**E**); Ca_V_2.1/β4/α_2_δ-2 (black bar), Ca_V_2.1/β4 alone (white bar) and Ca_V_2.1/β4/α_2_δ-2/WT PrP (grey bar). **P<0.01; NS, not significant. All statistical differences were determined by one-way ANOVA and Dunnett's multiple comparison test.

We then examined whether PrP would influence the ability of α_2_δ to increase calcium channel currents. We expressed Ca_V_2.1/β4 and α_2_δ-2 to mimic the calcium channel combination present in cerebellar Purkinje cells, either with or without PrP. We found that PrP co-expression produced a moderate, but consistent, reduction in peak calcium channel currents at +10 mV of approximately 34%, although the currents in the presence of PrP and α_2_δ remained significantly larger than those in the absence of α_2_δ (Figures 2A–2C). The voltage-dependence of activation of the Ca_V_2.1/β4/α_2_δ-2 currents in the presence of PrP was also significantly depolarized, compared with in its absence, although not to the same extent as in the absence of α_2_δ (Figure 2D). Similarly, the voltage-dependence of steady-state inactivation of Ca_V_2.2/β4/α_2_δ-2 was depolarized significantly in the additional presence of PrP (Figures 2E and 2F). All these effects are indicative of a reduced enhancement by α_2_δ-2 of Ca_V_2.1/β4 calcium channel currents when PrP was co-expressed.

### Co-expression of PrP decreased calcium channel currents containing Ca_V_2.1/β1b/α_2_δ-1

**Figure 3:**
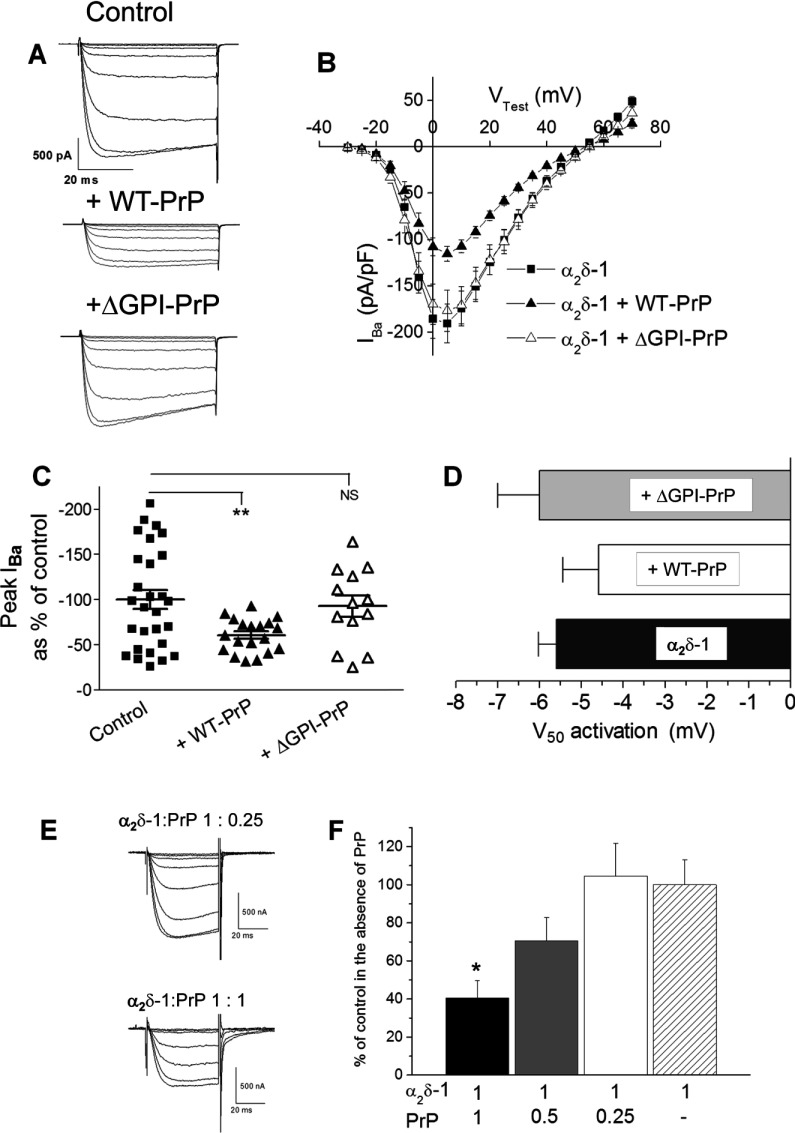
Effect of PrP constructs on Ca_V_2.1/β1b/α_2_δ-1 calcium channel currents (**A**) Examples of families of *I*_Ba_ current traces resulting from step potentials from −90 mV to between −30 and +10 mV in 5 mV steps for Ca_V_2.1/β1b/α_2_δ-1 alone (top panel) with WT PrP (middle panel) or with ΔGPI–PrP (bottom panel). The ratio of cDNAs used for the transfection of Ca_V_2.1/β1b/α_2_δ-1/PrP was 3:2:2:1, with empty vector used where α_2_δ or PrP was absent. (**B**) *I*–*V* relationships for *I*_Ba_ recorded from tsA-201 cells expressing Ca_V_2.1/β1b/α_2_δ-1 alone (■; *n*=28) with WT PrP (▲; *n*=19) or with ΔGPI–PrP (△; *n*=13). (**C**) Individual mean±S.E.M. peak *I*_Ba_ currents at +5 mV for Ca_V_2.1/β1b/α_2_δ-1 alone (■; *n*=28) with WT PrP (▲; *n*=19) or ΔGPI–PrP (△; *n*=13). **P<0.01. (**D**) *V*_50_ activation for Ca_V_2.1/β1b/α_2_δ-1 alone (black bar) with WT PrP (white bar) or with ΔGPI–PrP (grey bar). All statistical differences were determined by one-way ANOVA and Dunnett's multiple comparison test. (**E** and **F**) Ca_V_2.2/β1b/α_2_δ-1 was expressed in *Xenopus* oocytes either alone or together with PrP at the ratios shown. (**E**) Representative current traces elicited by steps to test potentials between −25 and +10 mV in 5 mV steps from a holding potential of −100 mV for Ca_V_2.2/β1b/α_2_δ-1/PrP (1:0.25; upper panel) or Ca_V_2.2/β1b/α_2_δ-1/PrP (1:1; lower panel). The residual voltage clamp transients have been truncated. (**F**) Peak currents measured at +10 mV for three α_2_δ-1/PrP ratios plotted as the mean±S.E.M. percentage of the mean control *I*_Ba_ recorded in the absence of PrP in the same experiment. α_2_δ-1/PrP 1:1 (black bar; *n*=6), α_2_δ-1/PrP ratio 1:0.5 (grey bar; *n*=18), α_2_δ-1/PrP ratio 1:0.25 (white bar; *n*=20) and mean normalized control for α_2_δ-1 in the absence of PrP (hatched bar; *n*=21). *P=0.016 between the individual conditions and their respective control condition performed in the same experiment as determined by Student's *t* test.

We then examined whether PrP would also inhibit calcium channel combinations containing α_2_δ-1, and tested whether any effect could be due to the demonstrated interaction at the level of the two polypeptides [35] or a result of lipid anchoring and co-localization in the same cholesterol-rich microdomains. We therefore expressed Ca_V_2.1/β1b/α_2_δ-1 with either WT PrP or anchorless PrP, truncated at the GPI-anchor site (ΔGPI–PrP) [38]. We observed that co-expression with WT PrP reduced the peak calcium channel currents to a similar extent (39%) to that shown for the Ca_V_2.1/β4/α_2_δ-2 combination (Figures 3A–3C), whereas ΔGPI–PrP had no effect on the Ca_V_2.1/β1b/α_2_δ-1 calcium channel currents (Figures 3A–3C). In these experiments WT PrP did not produce a significant depolarization of the voltage-dependence of activation of the currents (Figure 3D).

In another expression system, *Xenopus* oocytes with microinjected cDNAs and where the ratio of the different constructs can be controlled accurately, the effect of PrP to reduce Ca_V_2.2/β1b/α_2_δ-1 calcium channel currents was observed at an α_2_δ-1/PrP ratio of 1:1, but not at 2:1 or 4:1 (Figures 3E and 3F), indicating that it was dependent on the amount of PrP expressed relative to α_2_δ. Taken together, these results suggest that the effect of PrP to reduce calcium channel currents is concentration-dependent, and the presence of a GPI anchor on PrP may be essential for the observed reduction in the functional expression of calcium currents.

### Membrane anchoring of both PrP and α_2_δ-1 is required for calcium channel current inhibition by PrP

**Figure 4:**
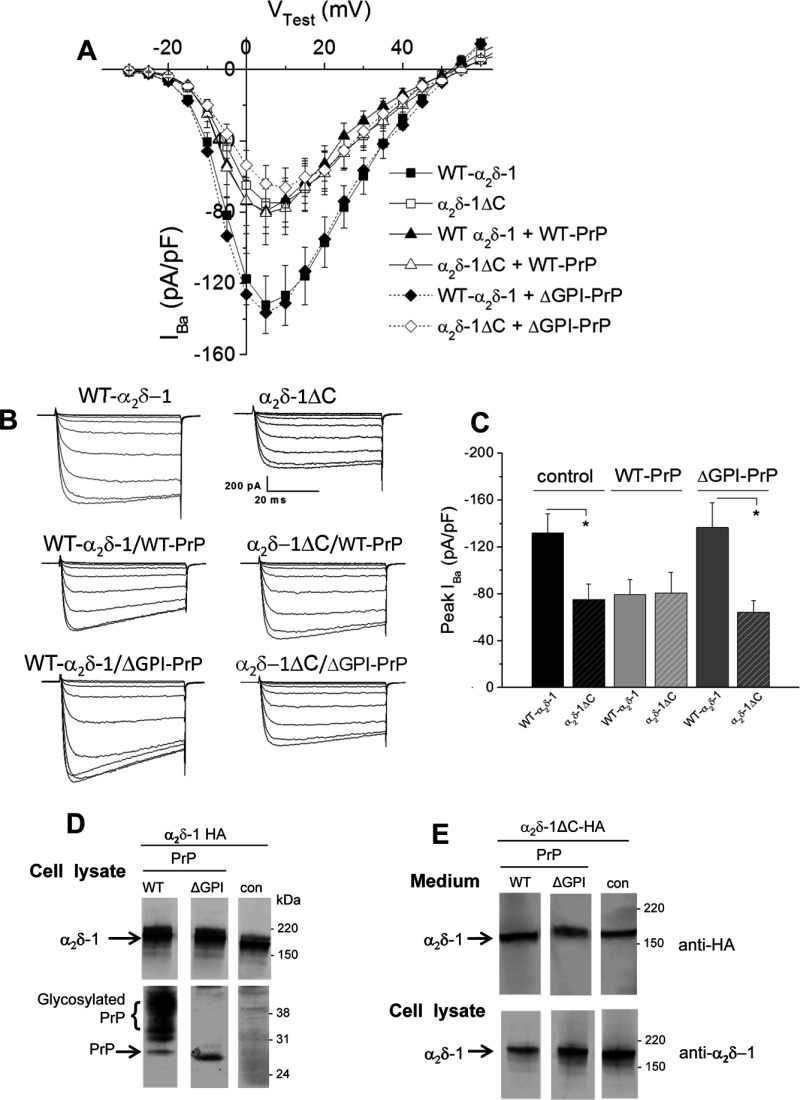
Comparison of the effect of PrP constructs on calcium channel currents containing α_2_δ-1 or anchorless α_2_δ-1 (**A**) *I-*-*V* relationships for *I*_Ba_ recorded from tsA-201 cells expressing Ca_V_2.1/β1b/α_2_δ-1 alone (■; *n*=16) with WT PrP (▲; *n*=10) or ΔGPI–PrP (◆; *n*=14) or cells expressing Ca_V_2.1/β1b/α_2_δ-1ΔC alone (□; *n*=11) with WT PrP (∆; *n*=13) or ΔGPI–PrP (◇; *n*=15). The ratio of cDNAs was the same as in Figure 3(A). (**B**) Examples of families of *I*_Ba_ current traces resulting from step potentials from −90 mV to between −30 and +10 mV in 5 mV steps for Ca_V_2.1/β1b/α_2_δ-1 alone (top left-hand panel) or α_2_δ-1ΔC alone (top right-hand panel) with WT PrP (middle panel) or ΔGPI–PrP (bottom panels). (**C**) Peak *I*_Ba_ currents (means±S.E.M.) for the data shown in (**A**) for Ca_V_2.1/β1b with α_2_δ-1 (solid bars) or with α_2_δ-1ΔC (hatched bars) either alone (black bars) or with WT PrP (light grey bars), or ΔGPI-PrP (dark grey bars). *P<0.05 as determined by one-way ANOVA and Bonferroni's post-hoc test. (**D**) Western blot of α_2_δ-1 (upper panels; 4–12% Bis-Tris gel) co-expressed (1:1) with WT PrP (left-hand lane), ∆GPI–PrP (middle lane) or empty vector (right-hand lane; con). PrP expression is shown in the lower panel (4–12% Bis-Tris gel). The lower expression of ∆GPI–PrP in the cell lysate is because much of it is secreted [53]. (**E**) Western blot of α_2_δ-1∆C in medium (upper panels; 3–8% Tris/acetate gel) and cell lysate (lower panels; 4–12% Bis-Tris gel) co-expressed (1:1) with PrP (left-hand panels), ∆GPI–PrP (middle panel) or empty vector (right-hand panels; con). In both (**D**) and (**E**), all three lanes are from the same blots from which irrelevant lanes have been excised and the molecular mass is given on the right-hand side in kDa.

In order to test the importance of membrane anchoring of both α_2_δ-1 and PrP on the inhibition by PrP we expressed Ca_V_2.1/β1b with either WT α_2_δ-1 or anchorless α_2_δ-1 (α_2_δ-1ΔC–HA), together with either WT PrP or anchorless PrP truncated at the GPI-anchor site (ΔGPI–PrP). We have shown previously that an anchorless form of α_2_δ-1, although being mainly secreted, is still able to promote an increase in calcium channel currents compared with no α_2_δ, although to a smaller extent than WT α_2_δ-1 [36]. In the present study we obtained the surprising result, shown in Figures 4(A)–4(C), that, whereas WT PrP inhibited calcium channel currents in the presence of WT α_2_δ-1, it did not produce any reduction in the presence of anchorless α_2_δ-1ΔC–HA (Figure 4C). In agreement with the data shown in Figure 3, ΔGPI–PrP did not produce any inhibition of calcium channel currents formed with WT α_2_δ-1 (Figure 4C). Similarly ΔGPI–PrP did not produce any reduction in the currents formed with anchorless α_2_δ-1ΔC–HA (Figure 4C). We found from Western blotting that the WT and truncated α_2_δ and PrP species used are all well-expressed in tsA-201 cells (Figures 4D and 4E), and lack of expression therefore could not account for the lack of effect of the truncated constructs when expressed together.

**Figure 5:**
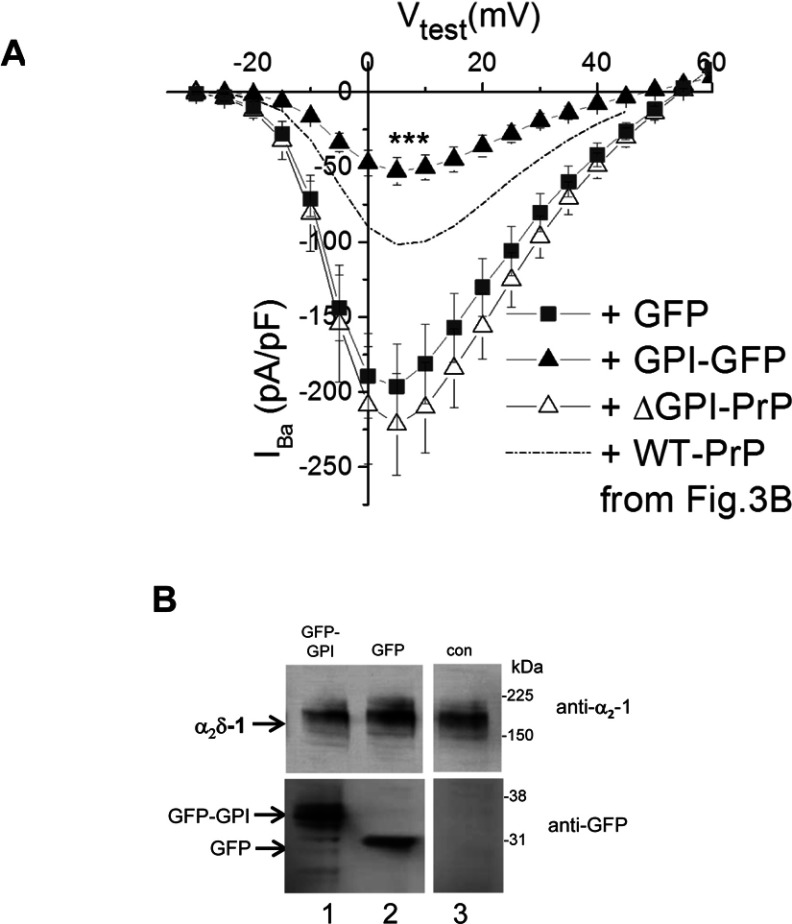
Effect of GPI–GFP on Ca_V_2.1/β1b/α_2_δ-1 calcium channel currents (**A**) *I*–*V* relationships for *I*_Ba_ recorded from tsA-201 cells expressing Ca_V_2.1/β1b/α_2_δ-1 with GFP (■; *n*=9), GPI–GFP (▲; *n*=9), or GFP and ΔGPI–PrP (△; *n*=6). The broken line indicates the level of *I*_Ba_ observed for Ca_V_2.1/β1b/α_2_δ-1 plus WT PrP from Figure 3(B). ***P<0.001 between the peak *I*_Ba_ when GPI–GFP was co-expressed compared with when GFP was co-expressed (Student's *t* test). (**B**) Western blot of α_2_δ-1 (upper panels) co-expressed (1:1) with GFP–GPI (lane 1), GFP (lane 2) or empty vector (lane 3; con). GFP expression is shown in the lower panel. All three lanes are from the same blots from which irrelevant lanes have been excised and the molecular mass is given on the right-hand side in kDa.

This result suggests either that association of the α_2_δ and PrP proteins in the plasma membrane, and potentially in the same cholesterol-rich membrane microdomains, facilitated by their GPI anchoring, is essential for the inhibition of calcium currents observed or that there is competition between α_2_δ-1 and PrP for the GPI-anchor intermediates, resulting in less maturation of α_2_δ subunit in the presence of PrP. We therefore examined whether the effect of PrP might be related to the GPI anchor and tested the effect of an unrelated protein engineered to contain a GPI anchor (GFP–GPI). We found that co-expression of GFP–GPI markedly reduced calcium channel currents formed by Ca_V_2.1/β1b/α_2_δ-1 compared with the co-expression of cytosolic GFP, and in this experiment ∆GPI–PrP again had no effect (Figure 5A). This result is in agreement with the hypothesis that overexpression of a GPI-anchored protein can produce this inhibition. We then compared the level of α_2_δ-1 expression in the presence of co-expressed GFP–GPI and cytosolic GFP (Figure 5B). In all cases substantial α_2_δ-1 was co-expressed.

## DISCUSSION

In the present study we found that when PrP is co-expressed with Ca_V_2.1 calcium channels together with a β-subunit and either α_2_δ-1 or α_2_δ-2, it produces a modest reduction in the expressed calcium channel currents. This reduction in calcium channel currents involves the membrane anchoring of PrP, as it is absent when PrP is truncated prior to its GPI-anchor site. Thus ΔGPI–PrP did not inhibit currents formed by the expression of Ca_V_2.1/β1b/α_2_δ-1. This result suggests that the process might involve competition for pathways associated with the GPI anchoring of PrP, which might relate to our finding that α_2_δ subunits form GPI-anchored proteins [13]. The results of the present study reinforce that view, since native cerebellar α_2_δ-1, α_2_δ-2 and PrP all behave similarly following PI-PLC treatment and phase separation, being concentrated in the aqueous phase (Figure 1), which is supportive of evidence for GPI anchoring.

We therefore utilized an α_2_δ-1 subunit truncated at the predicted membrane attachment site (α_2_δ-1ΔC), which, as we have shown recently, is not an integral membrane protein [36]. We have characterized α_2_δ-1ΔC and found that, despite being mainly secreted, it still shows a partial ability to enhance calcium channel currents and to be associated extrinsically with the plasma membrane [36]. It is therefore possible that α_2_δ-1 enhances calcium currents by two mechanisms, one involving a trafficking process that is still maintained by anchorless α_2_δ-1∆C, and another mechanism that requires its membrane anchoring, for example a process that stabilizes channel complexes in the plasma membrane.

Importantly, we also found in the present study that WT PrP does not inhibit calcium channel currents when α_2_δ-1ΔC was substituted for WT α_2_δ-1. This suggested to us two possibilities. First, PrP and α_2_δ-1 may compete for GPI-anchor intermediates or subsequent trafficking processes specific for GPI-anchored proteins when both are overexpressed. Secondly, the interaction between PrP and α_2_δ-1 with other protein and lipid components in cholesterol-rich membrane microdomains [50,51] might be involved in the suppression of currents. Thus when either PrP or α_2_δ-1 is devoid of the lipid anchor no suppression occurs.

It is possible that WT α_2_δ-1 and PrP interact indirectly via an intermediary trafficking protein and compete for binding to that protein, whereas this does not occur when one of them is not membrane-anchored. Nevertheless, the additional finding that Ca_V_2.1/β1b/α_2_δ-1 currents were also inhibited by GPI–GFP (when compared with the control GFP) suggests that the presence of a GPI anchor might be an important common denominator in this process. Only approximately 150 proteins are known to be GPI-anchored, and the GPI synthetic pathway, involving multiple enzymatic steps in the ER (endoplasmic reticulum) and Golgi, is likely to have limited capacity in mammalian cells [52]. Overexpression of multiple GPI-anchored proteins in the same cell might lead to saturation of limited resources. Further studies will be necessary to determine whether overexpression of other GPI-anchored proteins disrupts the function of α_2_δ within membrane microdomains, or possibly competes with it for GPI transamidase enzymes, GPI-anchoring intermediates or trafficking proteins subsequent to GPI anchoring. Taken together, the results of the present study provide supporting evidence that α_2_δ-1 can be GPI-anchored. More generally the results point to a competitive interaction between GPI-anchored proteins for resources that determine their level of post-translational processing or expression on the plasma membrane.
